# PET Imaging of Microtubules in Cognitively Impaired and Normal Older Adults

**DOI:** 10.1002/alz.095392

**Published:** 2025-01-09

**Authors:** Naresh Damuka, Mack Miller, Ivan Krizan, Bhuvanachandra Bhoopal, Miranda E. Orr, Krishna Kumar Gollepalli, Samuel N. Lockhart, Christopher T Whitlow, Akiva Mintz, Suzanne Craft, Kiran K. Solingapuram Sai

**Affiliations:** ^1^ Wake Forest University School of Medicine, Winston‐Salem, NC USA; ^2^ Columbia University Medical Center, New York, NY USA

## Abstract

**Background:**

Although associated with early changes in Aβ and tau formations, microtubule (MT) destabilization has not yet been systematically evaluated as an early biomarker of neurodegeneration and a tool to quantify it in real‐time could provide information on these early changes in AD cascade. While current Aβ and tau PET approaches can characterize disease lesions, they fall short in capturing earlier molecular events. Our team development the first brain‐penetrant MT PET ligand, [^11^C]MPC‐6827. We evaluated its *in vivo* imaging efficacy in rodent and nonhuman primate models of AD, and subsequently conducted a first‐in‐human study. Here we show our preliminary MT‐PET and autoradiography data in cognitively impaired and age‐matched cognitively normal older adults and post‐mortem human brains respectively.

**Method:**

Dynamic 1h brain PET imaging was conducted with [^11^C]MPC‐6827 in cognitively impaired (Aβ and tau PET positive) and cognitively normal (Aβ and tau PET negative) older adults (n = 5/group). MT‐PET uptake was correlated with [^11^C]PiB‐Aβ, and FTP‐tau SUVRs and fluid biomarker data. Autoradiography was performed in postmortem MCI/early AD (n = 3) and age‐matched cognitively normal adults (n = 2) brain tissues.

**Result:**

[^11^C]MPC‐6827 uptake was higher in the frontal and prefrontal cortex, hippocampus, putamen, and pons of the cognitively impaired subjects than the controls. Uptake correlated positively with ptau181, [^11^C]PiB Aβ, and FTP‐tau SUVRs and inversely with their CSF Aβ_42_—validating its relevance to AD biomarkers. MT‐PET‐SUVRs positively correlated with calculated average PiB SUVRs (Pearson *r* = 0.667, *p* = 0.05) and with FTP SUVRs (*r* = 0.367, ^*^ *p* = 0.047). Autoradiography uptake in AD prefrontal cortex and hippocampus was ∼39 and ∼43% (*p≤0.05) higher than that in control tissues.

**Conclusion:**

Our [^11^C]MPC‐6827 human studies demonstrate (a) safety and feasibility; (b) robust brain uptake; (c) higher brain uptake in cognitively impaired subjects than in controls; (d) a highly positive correlation among PiB, and FTP uptakes; (e) autoradiography results may indicate not possibly of age confounding the imaging data. This study will be the first to image MT destabilization processes in AD. It will create a new understanding of MT‐based processes in the neurodegeneration cascade to optimize approaches of AD diagnosis and monitoring.

